# Supplementary material for: Compritol-Based Alprazolam Solid Lipid Nanoparticles for Sustained Release of Alprazolam: Preparation by Hot Melt Encapsulation
Source: Molecules. 2022 Dec 14;27(24):8894. doi: 10.3390/molecules27248894 (PMC9783086; doi:10.3390/molecules27248894)
Supplement: Supplementary file 1 [file molecules-27-08894-s001.zip › molecules-2045269-supplementary.pdf]

Supplementary Materials

Compritol-Based Alprazolam Solid Lipid Nanoparticles for Sustained Release of Alprazolam: Preparation by Hot Melt Encapsulation

Huma Rao [1,*], Saeed Ahmad [1], Asadullah Madni [2], Iqra Rao [3], Mohammed Ghazwani [4], Umme Hani [4], Muhammad Umair [5,*], Imtiaz Ahmad [1], Nadia Rai [2], Maqsood Ahmed [1] and Kashif ur Rehman Khan [1,*]

1 Department of Pharmaceutical Chemistry, Faculty of Pharmacy, The Islamia University of Bahawalpur, Bahawalpur 63100, Pakistan
2 Department of Pharmaceutics, Faculty of Pharmacy, The Islamia University of Bahawalpur, Bahawalpur 63100, Pakistan
3 Department of Community Medicine, King Edward Medical University Lahore, Lahore 54000, Pakistan
4 Department of Pharmaceutics, College of Pharmacy, King Khalid University, Abha 62529, Saudi Arabia
5 College of Pharmacy, Shenzhen Technology University, Shenzhen 518060, China
* Correspondence: huma.rao@iub.edu.pk (H.R.); umair_uaf@hotmail.com (M.U.); kashifur.rahman@iub.edu.pk (K.u.R.K.); Tel: +49-5117625589

Table S1. Individual particle size determination of AF6 from SEM images by Image J sizer.

Selection Area	Particle size (nm)
1	100
2	117.39
3	117.39
4	104.35
5	126.09
6	182.61
7	180
8	147.83
9	195.65
10	130.43
11	186.96
Average Particle Size	144.42
